# Validation of an algorithm to predict decline in INR following warfarin cessation in patients undergoing invasive procedures

**DOI:** 10.1007/s11239-019-02017-2

**Published:** 2019-12-17

**Authors:** Emmanouela Kampouraki, Hilary Wynne, Peter Avery, Farhad Kamali

**Affiliations:** 1 Translational and Clinical Research Institute, Newcastle University, Newcastle upon Tyne, UK; 2 Older People’s Medicine, Freeman Hospital, Newcastle upon Tyne, UK; 3 School of Mathematics Statistics and Physics, Newcastle University, Newcastle upon Tyne, UK; 4 Translational and Clinical Research Institute, Newcastle University and Newcastle upon Tyne Hospitals, NHS Foundation Trust, Newcastle upon Tyne, NE1 7RU UK

**Keywords:** Warfarin, International normalized ratio, Algorithm, Cytochrome P-450 CYP2C9, Genotype

## Abstract

Patients on warfarin are required to withdraw from treatment for a fixed period (normally 5 days) prior to an invasive procedure. However, the anticoagulant effect of warfarin subsides at different rates among different patients, exposing some to increased risk of either thrombosis or bleeding. In a recent study in patients awaiting surgery, following warfarin cessation the INR declined slower over time in those with two *CYP2C9* variant alleles, increasing age, weight and number of comorbidities and that INR decline was faster in those with higher maintenance INR value. Subsequently, we developed an algorithm which predicts INR decline in individual patients after 5 days of warfarin cessation. The current study validated the algorithm. An independent cohort of patients completing a short course of warfarin took part in the study. INR values for subsequent 9 days and *CYP2C9* genotype were available. The predicted INR decline (INR_day 1_–INR_day 5_) was compared to the observed one (where an INR check on day 5 was unavailable, INR was estimated using a linear approximation model). There was a strong correlation between the decline in INR by day 5 and that predicted from the algorithm for the 117 patients (r = 0.949, p < 0.001). The algorithm was precise, with low degree of bias and variance of the prediction error. The algorithm can accurately predict the INR decline following warfarin cessation in individual adult patients. The use of this easily adoptable algorithm can reduce cancellation or delays of planned surgical procedures.

## Highlights

- The effect of warfarin subsides at different rates among different patients based upon individual patient characteristics.
- An algorithm predicting the fall in INR within 5 days in individual patients was validated.
- There was a strong and significant correlation between the observed and predicted fall in INR.
- The validated algorithm can accurately predict the fall in INR after warfarin cessation for individual patients and is easy to implement in clinic.

## Introduction

Patients on warfarin are required to stop treatment for a fixed period (5 days according to local guidelines) prior to an invasive procedure, in order to minimise the risk of peri-operative bleeding [1]. However, the anticoagulant activity of warfarin subsides at different rates among different patients, thus withholding warfarin for 5 days may not result in restoration of normal coagulation in all patients. Discontinuation of therapy too early may predispose the patient to thrombosis or stopping it too late may result in cancellation of the planned procedure due to risk of peri-operative bleeding.

In a recent study in adult patients who interrupted warfarin therapy prior to elective surgery, we demonstrated that the rate of INR decline following warfarin cessation was slower in patients with two *CYP2C9* variant alleles, increasing age, weight and number of comorbidities and faster with greater INR value prior to warfarin withdrawal. These factors accounted for approximately 90% of the inter-individual variability in the rate of INR decline [2]. Based upon the study results an algorithm was developed to predict the fall in INR in individual patients 5 days after warfarin cessation.

This study aimed to validate the algorithm, using data from an independent cohort of patients who had discontinued warfarin therapy for a reason unrelated to surgery.

## Patients, materials and methods

### Study cohorts

The algorithm developed was derived by multiple regression analysis of data in a cohort of adult patients recruited as part of an earlier study [2] (designated as the algorithm cohort) as shown below:

$$INR\;decline\;by\;day\;5 = - 0.195 - 0.00428\;\left\{ {age} \right\} - 0.2374\;\left( {n.CYP2C9} \right) + 0.9143\;\left\{ {INR} \right\} - 0.00246\;\left\{ w \right\} - 0.0306\;\left( {n.com} \right)$$

where age in years; n.CYP2C9: presence of CYP2C9 double variant (either *3*3, *3*2, or *2*2) = 1 and absence of double variant = 0, index INR (INR on day 1); w: weight in kg and n.com: number of comorbidities, including AF or other indication for warfarin therapy.

The inclusion and exclusion criteria of patients in the algorithm cohort have been previously reported [2].

Inclusion criteria for patients used to validate the algorithm (designated as the validation cohort) were the ability to provide informed consent, age ≥ 18 years, and about to discontinue warfarin therapy. Patients were excluded if they were either taking any concurrent medication or had any chronic condition that may affect warfarin disposition or its pharmacologic activity, or excessive alcohol intake that could have affected anticoagulation response to warfarin.

### Data analysis

Data belonging to an independent cohort of Caucasian patients aged ≥ 18 years, who had completed a course of warfarin, were available for the algorithm validation. Information on demographics, clinical data, *CYP2C9* genotype and INR value (measured in citrated plasma specimens) on the day of warfarin cessation and on alternate days for the following 9 days were available [3].

The accuracy of the algorithm was tested by assessing the correlation between the observed INR decline [calculated by subtracting the INR value on day 5 from the index INR value in the morning following warfarin cessation (day 1)] and the predicted decline in INR according to the algorithm. The performance of the algorithm was evaluated, according to the method of Sheiner and Beal [4], using the root mean squared prediction error (RMSE) as a measure of precision and the mean prediction error (ME) as a measure of bias. The mean squared deviation of prediction errors from their mean (mSDEM) was determined as an estimate of the variance of the prediction error.

The days of INR checks following warfarin cessation varied among the validation cohort for scheduling purposes. For patients with an INR check on day 5 (± 10 h) the INR value was designated as the observed value. To determine the INR value on day 5 for those without an INR check on that day, a linear approximation model that assumes an exponential INR decline asymptotically over time was used as previously described by White and colleagues [5] with a further assumption that over time INR reaches a baseline value of 0.8 [6]. Transformation of INR into logarithm after subtracting the asymptote results in a linear model. The INR value on day 5 was derived from the plot of the natural logarithm of INR-0.8 against time in hours following warfarin cessation.

Excel (Microsoft Corp., Redmond, WA, USA) was used for data collation and Minitab (Coventry, UK) was used for statistical analysis. Where necessary, data were transformed to achieve approximate normality. Demographic and clinical data common to both cohorts were used for comparison. Unless otherwise stated, INR decline values are presented as mean (range).

## Results

Of the 131 patients available in the validation cohort, 14 were excluded from final analysis for the following reasons; 7 had an index INR of 1.5 or lower, 2 had no index INR value recorded, 2 had data missing on at least two parameters included in the algorithm and 3 had their last recorded INR check > 40 h before day 5. Overall, 117 patients were available for analysis, with mean index INR value of 2.5. Demographics for both patient cohorts are presented in Table 1. Patients in the algorithm and validation cohorts were significantly different in age (p < 0.001 for males and p = 0.002 for females; Student’s t-test), mean warfarin weekly dose (p = 0.004 for males and p = 0.036 for females; Student’s t-test) and indication for anticoagulation (p < 0.001; Chi square test).

**Table 1:** Patient demographics for the algorithm and validation cohorts

	Algorithm cohort	Validation cohort	p value
No of patients	152	117	
Sex, n (%)			^g^
Male	102 (67)	65 (55.6)	0.053
Female	50 (33)	52 (44.4)	
Age (years), mean (range)		^a^	^f^
Male	74 (43–90)	63 (29–92)	< 0.001
Female	72 (49–93)	64 (21–88)	0.002
Height (cm), mean ± SD	^e^	^c^	^f^
Male	174 ± 9	176 ± 7	0.052
Female	160 ± 5	161 ± 8	0.224
Weight (kg), mean ± SD		^c^	^f^
Male	88 ± 17	92 ± 20.3	0.081
Female	81 ± 19	81 ± 22.6	1.000
Warfarin weekly dose (mg), mean ± SD		^d^	^f^
Male	28.7 ± 10.3	34.9 ± 15.0	0.004
Female	27.4 ± 14.0	33.4 ± 14.5	0.036
Indication for anticoagulation, n (%)			^g^
Atrial fibrillation	125 (82)	12 (10.3)	< 0.001
Venous thromboembolism	27 (18)	63 (53.8)	
Pulmonary embolism	0	33 (28.2)	
Other	0	9 (7.7)	
No of comorbidities, mean (range)^b^	3 (1–8)	2 (1–6)^c^	< 0.001
No of concomitant medications, mean (range)	5 (1–12)	5 (1–15)	0.093
*CYP2C9* genotype		^d^	^g^
*1/*1	92	64	0.553
*1/*2	35	31	
*1/*3	15	14	
*2/*2	4	5	
*2/*3	6	1	
*3/*3	0	0	
Index INR mean ± SD (range)	–	2.5 ± 0.6 (1.6–4.5)	

^a^Based on 112 patients

^b^The reported number of comorbidities includes indication for anticoagulation and the number of concomitant medications includes warfarin

^c^Based on 116 patients

^d^Based on 115 patients

^e^Based on 140 patients

^f^Student’s t-test

^g^Chi squared test

All but one patients reached INR < 1.5 at day 5. A single patient with INR = 1.6 at day 5, which was a predicted value through the linear approximation model in the absence of an INR check close to day 5, was a 71-year old female, weighing 94 kg, with index INR of 2.3, 2 comorbidities and carrying one variant *CYP2C9* allele.

Seventy patients had an INR check on day 5 (± 10 h). The observed INR on day 5 was 1.1 (0.9–1.4). The decline in the observed INR value (i.e. INR on day 1 − INR on day 5) was 1.4 (0.4–3.5) while the algorithm predicted a decline in INR of 1.6 (0.9–3.4). The actual and predicted INR declines were strongly and significantly correlated (r = 0.969, p < 0.001; Pearson test).

**Fig. 1:**
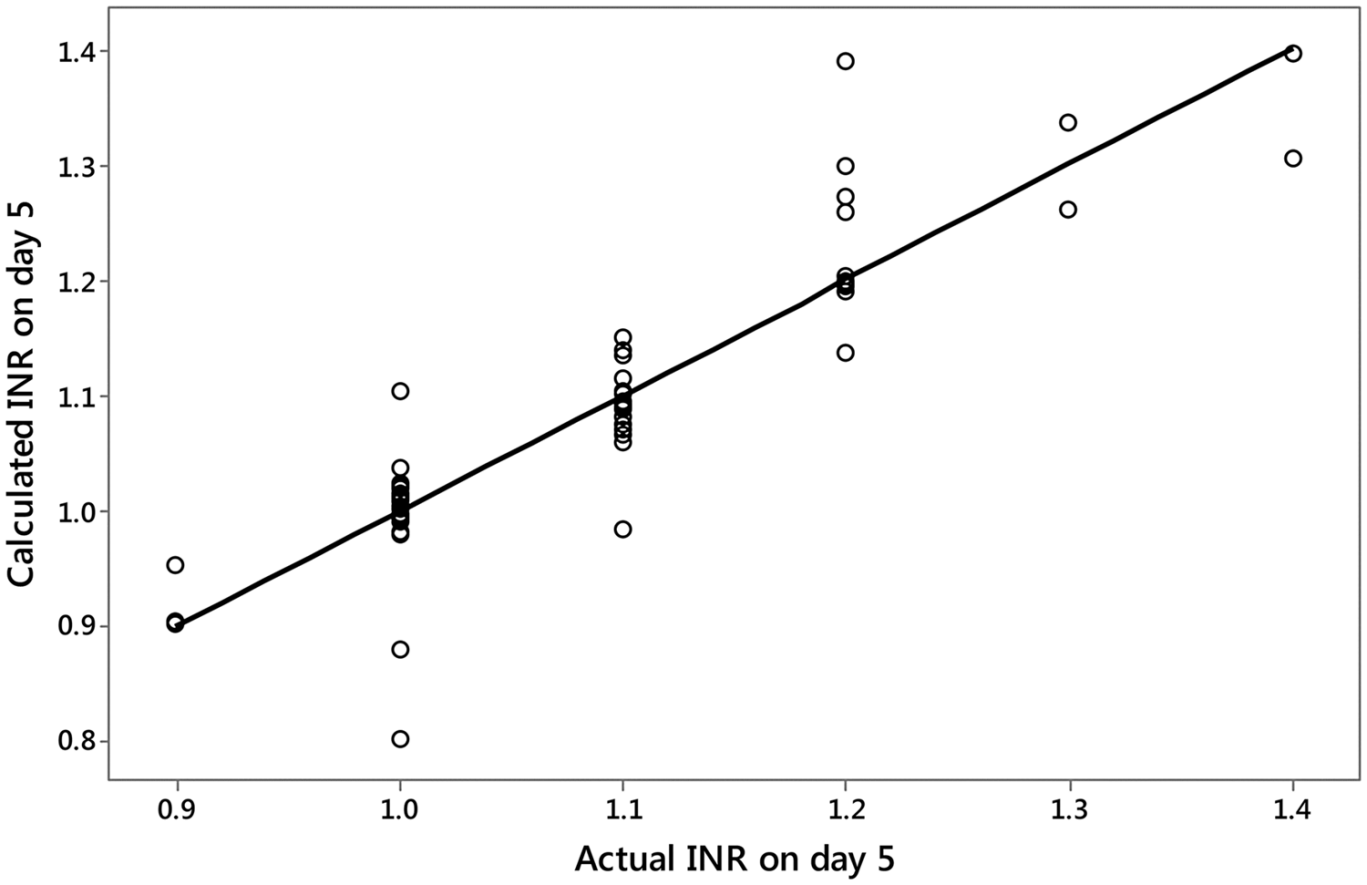
Actual INR value on day 5 following warfarin cessation versus INR value obtained from linear regression slope (n = 70)

To confirm the accuracy of the linear approximation model the INR value on day 5 was estimated according to the model, as explained earlier, and the estimated INR values were compared to the actual values in the above subgroup of patients. There was a very strong and highly significant correlation (r = 0.905, p < 0.001; Spearman test, Fig. 1) between the actual INR and estimated INR values, with a maximum difference in INR of 0.2 units, which is not clinically significant.

For 47 patients INR check on day 5 was not recorded. Therefore, INR on day 5 was estimated using the linear approximation model, with a mean of 1.1 (0.9–1.6) and a decline in INR (INR on day 1–INR on day 5) of 1.3 (0.5–3.3). Based on the algorithm, the predicted decline in INR is 1.5 (0.6–3.3). There was a strong and highly significant correlation between the decline in the INR derived from the model and that predicted from the algorithm (r = 0.932, p < 0.001; Spearman test).

**Fig. 2:**
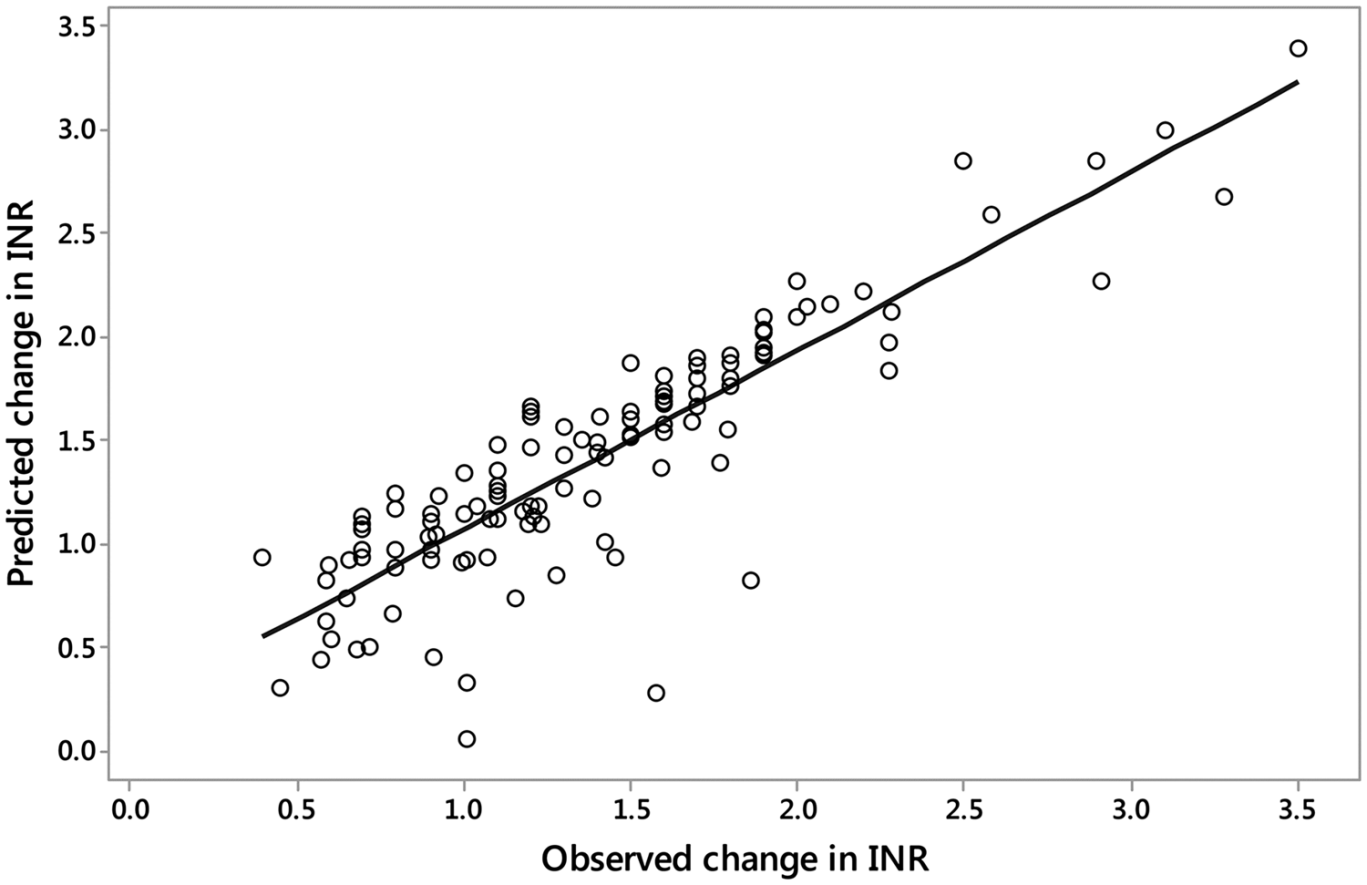
Predicted versus observed change in INR (day 1–day 5)

Overall, there was a strong and highly significant correlation between the decline in INR by day 5 (based on both actual INR value and that predicted from the linear approximation model) and that predicted from the algorithm for the whole cohort of 117 patients (r = 0.949, p < 0.001; Spearman test, Fig. 2). The RMSE was 0.22, indicating that the algorithm was precise at 0.22 units of INR decline. The ME was 0.15 and the mSDEM was 0.026, which confirm the low degree of bias and variance of the prediction error.

Of the whole validation cohort, only 6 patients (5.1%) had double variant *CYP2C9* genotype which is consistent with the observed low frequency of this genotype in the Caucasian population [7]. Removing the effect of genotype from the algorithm resulted in an increase of 0.2 units in the predicted INR decline for these patients [0.1 (0.0–0.2)]. Overall, for all 117 patients and after removing the effect of genotype from the algorithm, there was a strong and highly significant correlation between the observed and predicted decline in INR (r = 0.958, p < 0.001; Spearman test) and equal predictive performance (RMSE = 0.22, ME = 0.16 and mSDEM = 0.022).

For greater ease of use, the algorithm can be transformed to a more simplified version as follows:

$$INR\;decline\;by\;day\;5 = 0.9\;\left\{ {INR} \right\} - 0.2\;\left\{ {N.CYP2C9} \right\} - 0.2 - \left[ {13\;\left\{ {AGE} \right\} + 7.4\;\left\{ W \right\} + 92\;\left\{ {N.COM} \right\}} \right]/3000$$

where INR; index INR, N.CYP2C9; the presence of CYP2C9 double variant (either *3*3, *3*2, or *2*2) = 1 and absence of double variant = 0, AGE; age in years, W; weight in kg, N.COM; number of comorbidities.

This simplified algorithm has comparable accuracy to the original algorithm in terms of high precision (RMSE = 0.19), low degree of bias (ME = 0.11) and small variance of the prediction error (mSDEM = 0.026).

## Discussion

Approximately 1 in 6 patients per year treated with warfarin undergo invasive surgical procedure and are required to discontinue treatment prior to the event [8]. In order to minimize the risk of perioperative bleeding, guidelines suggest that warfarin therapy is stopped 5 days before an invasive procedure [1, 9–11]. This recommendation is based upon the estimated warfarin clearance and the rate of production of functional coagulation factors II and X after the withdrawal of warfarin treatment [12]. However, a significant proportion of patients awaiting surgery remain above the INR threshold of 1.5 five days after the cessation of warfarin therapy [5, 13, 14] which necessitates the cancellation or delay of the planned procedure. This can be costly to healthcare providers and inconvenient to the patient.

An accurate method of predicting INR decline in individual patients is particularly useful for those with extreme demographics and in frail older patients with many comorbidities and other physiological factors that affect normal coagulation, who may present with therapeutic INR on the day of surgery [15, 16]. Accurate prediction of INR is also relevant for people withdrawing from warfarin with the aim of switching to treatment with DOACs, in which case there is specific threshold of INR value below which the transitioning to a DOAC can be performed (when INR is ≤ 3.0 for rivaroxaban, ≤ 2.5 for edoxaban, and ≤ 2.0 for apixaban and dabigatran) [17].

Warfarin elimination is significantly influenced by polymorphisms in the *CYP2C9* gene, which reduce the ability of the CYP2C9 enzyme to metabolise S-warfarin, as well as other demographic characteristics such as age, which influences both liver size and liver blood flow [18]. Earlier, our group demonstrated that the variability in INR decline following withdrawal from warfarin therapy is influenced by *CYP2C9* genetic polymorphism [3]. Our research group also more recently demonstrated that the variance in the rate of INR decline among patients following warfarin withdrawal is affected by polymorphism in *CYP2C9* gene (but polymorphisms in the *VKORC1* gene had no effect), as well as demographic and clinical factors [3]. A pharmacogenetic-guided algorithm was subsequently developed which could predict the INR decline for individual patients to reach < 1.5. The algorithm includes information on patient genetics (presence of *CYP2C9* double variant genotype), demographic (age and weight) and clinical data (index INR, and number of comorbidities).

In search of the clinical applicability of this algorithm, we tested its accuracy in a second independent cohort of patients on stable maintenance therapy withdrawing from a short course of warfarin treatment. The results showed that the predicted decline in INR on day 5 closely correlated with the observed decline in INR value. There was a slight over-estimation of the fall in INR (by 0.2 units) in the subgroup of 70 patients who had an INR check on day 5, and a slight under-estimation of INR in the subgroup of 47 patients who did not have an INR check on day 5. However, this is regarded to be within the expected variability and would not be clinically relevant, but would necessitate another INR check prior to an invasive procedure.

The two cohorts (algorithm and validation) were significantly different in a number of demographics and clinical data. The validation cohort included on average younger patients with a higher weekly dose of warfarin and greater variability in indication for anticoagulation. While these differences could affect the present study outcome, such variability is representative of the wide range of different populations discontinuing warfarin prior to an invasive procedure.

All but one patient in the validation cohort reached INR ≤ 1.5 on or before day 5. Therefore, the results of this algorithm validation study are applicable to patients that will reach INR of 1.5 or lower on day 5 after warfarin discontinuation.

Where INR data on day 5 were missing for some patients due to scheduling arrangements, the linear approximation model [5] was shown to be highly accurate in predicting the INR value in the group of patients with an actual INR check at or very close to day 5. The results of the present study are in agreement with the well-recognised association of *CYP2C9* genotype and age with the decline in INR following warfarin cessation, however there are also other studies which found no such associations, possibly because of missing data [19].

Considering the lack of genotyping facilities in many healthcare systems worldwide, this study also looked at the predictive ability and accuracy of the algorithm without the *CYP2C9* genotype component. The results showed that the predicted difference in INR decline (i.e. the difference between the INR decline predicted from algorithm containing *CYP2C9* genotype and that without genotype information) has only a minor effect on the predicted fall in INR and would be of limited clinical importance for the patient population in this study. The simpler version of the algorithm is readily adoptable for routine clinic use to predict the fall in INR in individual patients following warfarin cessation with or without information on patient *CYP2C9* genotype.

In conclusion, a more accurate prediction of the decline in INR could reduce the postponement or cancellation of planned invasive procedures and therefore be a safer and cost-effective way of withdrawing patients from warfarin prior to planned invasive procedure compared to the current practice. The present algorithm is best tailored to adult patients interrupting warfarin for an invasive procedure. There is no data available as to whether the algorithm is predictive of INR decline in patients who receive any medication, with excess alcohol intake or suffer from a medical condition that may affect warfarin pharmacology or anticoagulation response. Further study in patient populations discontinuing warfarin therapy, either specifically for a surgical procedure or for a different reason, with a greater proportion of patients remaining above the INR threshold of 1.5 at day 5 is necessary for the true clinical benefit of this algorithm to be appreciated.
